# Mendelian randomization reveals causal effects of kidney function on various biochemical parameters

**DOI:** 10.1038/s42003-022-03659-4

**Published:** 2022-07-18

**Authors:** Sehoon Park, Soojin Lee, Yaerim Kim, Semin Cho, Hyeok Huh, Kwangsoo Kim, Yong Chul Kim, Seung Seok Han, Hajeong Lee, Jung Pyo Lee, Kwon Wook Joo, Chun Soo Lim, Yon Su Kim, Dong Ki Kim

**Affiliations:** 1 Department of Biomedical Sciences, Seoul National University College of Medicine, Seoul, Korea; 2 Department of Internal Medicine, Uijeongbu Eulji University Medical Center, Gyeonggi-do, Korea; 3 Department of Internal Medicine, Seoul National University College of Medicine, Seoul, Korea; 4 Department of Internal Medicine, Keimyung University School of Medicine, Daegu, Korea; 5 Department of Internal Medicine, Chung-Ang University Gwangmyeong Hospital, Gyeonggi-do, Korea; 6 Department of Internal Medicine, Inje University Busan Paik Hospital, Busan, Korea; 7 Transdisciplinary Department of Medicine & Advanced Technology, Seoul National University Hospital, Seoul, Korea; 8 Department of Internal Medicine, Seoul National University Hospital, Seoul, Korea; 9 Kidney Research Institute, Seoul National University, Seoul, Korea; 10 Department of Internal Medicine, Seoul National University Boramae Medical Center, Seoul, Korea

**Keywords:** Nephrology, Diagnostic markers

## Abstract

The kidney is a vital organ with diverse biological effects and the burden of kidney function impairment is increasing in modern medicine. As the effects from kidney function on diverse biochemical parameters are yet fully understood, additional investigation to reveal the causal effects is warranted. Here we show the causal estimates from kidney function parameter, estimated glomerular filtration rate (eGFR), on 60 biochemical parameters by performing two-sample Mendelian randomization (MR) study in 337,138 white British UK Biobank participants. A higher genetically predicted eGFR was significantly associated with higher lymphocyte percentage, HDL cholesterol, and alanine aminotransferase. The causal estimates indicated that a higher genetically predicted eGFR was associated with lower urea, urate, insulin growth factor-1, and triglycerides levels. The parameters with significant but non-linear causal estimates were hemoglobin concentration, calcium, vitamin D, and urine creatinine values, identified by non-linear MR. Healthcare providers should understand that changes in eGFR may affect the identified biochemical parameters in diverse patterns. Future study is warranted to expand the knowledge of the mechanisms and clinical implications of the causal effects of eGFR on various biochemical parameters.

## Abstract (web summary)

Mendelian randomization analyses of estimated glomerular filtration rate (eGFR) in the UK Biobank cohort provide insight into the causal effects of kidney function on 60 biochemical parameters.

## Introduction

The kidney is a vital organ regulating volume homeostasis, toxin removal, electrolyte imbalance, and a variety of other biological processes. Chronic kidney disease (CKD), a state of impaired kidney function, is an increasingly prevalent comorbidity that imposes a large socioeconomic burden^1^. As kidney function is associated with diverse health outcomes, including death and cardiovascular events, this physiological variable is assessed frequently in current medical practice, commonly by measuring the estimated glomerular filtration rate (eGFR)^2^.

Observational studies have reported a number of biochemical abnormalities that are common in CKD patients, and there have been studies indicating that some laboratory findings (e.g., hyperuricemia) may increase the risk of CKD^3^. Conversely, as kidney clearance or related biological effects may affect biochemical assay results, biochemical imbalances common in CKD patients may be a result rather than a cause of impaired kidney function. In addition, although decreased kidney function is commonly defined as eGFR < 60 mL/min/1.73 m^2^, eGFR levels above the conventional cutoff may also have clinical importance, particularly considering what has been found regarding kidney hyperfiltration^4^. However, the causal effects of kidney function on biochemical abnormalities have been difficult to determine through observational studies due to the remaining possibility of reverse causation or effects of confounders. Knowing which biochemical parameters are causally affected by eGFR would aid in the interpretation of this frequently measured kidney function parameter and further the understanding of biological consequences related to the kidneys.

Mendelian randomization (MR) is an analytic tool that primarily focuses on investigating causal estimates of modifiable exposures to complex outcomes^5^. As genotypes are fixed at conception, genetically predicted exposure in MR is minimally affected by confounding effects or reverse causation, enabling a causal inference. MR has been implemented in studies investigating the causal estimates of complex traits (e.g., psychological phenotypes) on kidney function^6,7^. Furthermore, it is feasible to reverse the direction of MR analysis and use it to study the effects of kidney function on other phenotypes, as kidney function itself is explained by genetic information to a certain degree^8–10^.

In the present study, we aimed to establish causal estimates for the effects of eGFR on 60 biochemical phenotypes in the UK Biobank dataset through an MR analysis. We found that kidney function traits were causally linked to certain biochemical parameters in diverse patterns, which may expand the current understanding of the biological effects of kidney function.

## Results

### Characteristics of the study population

**Fig. 1:**
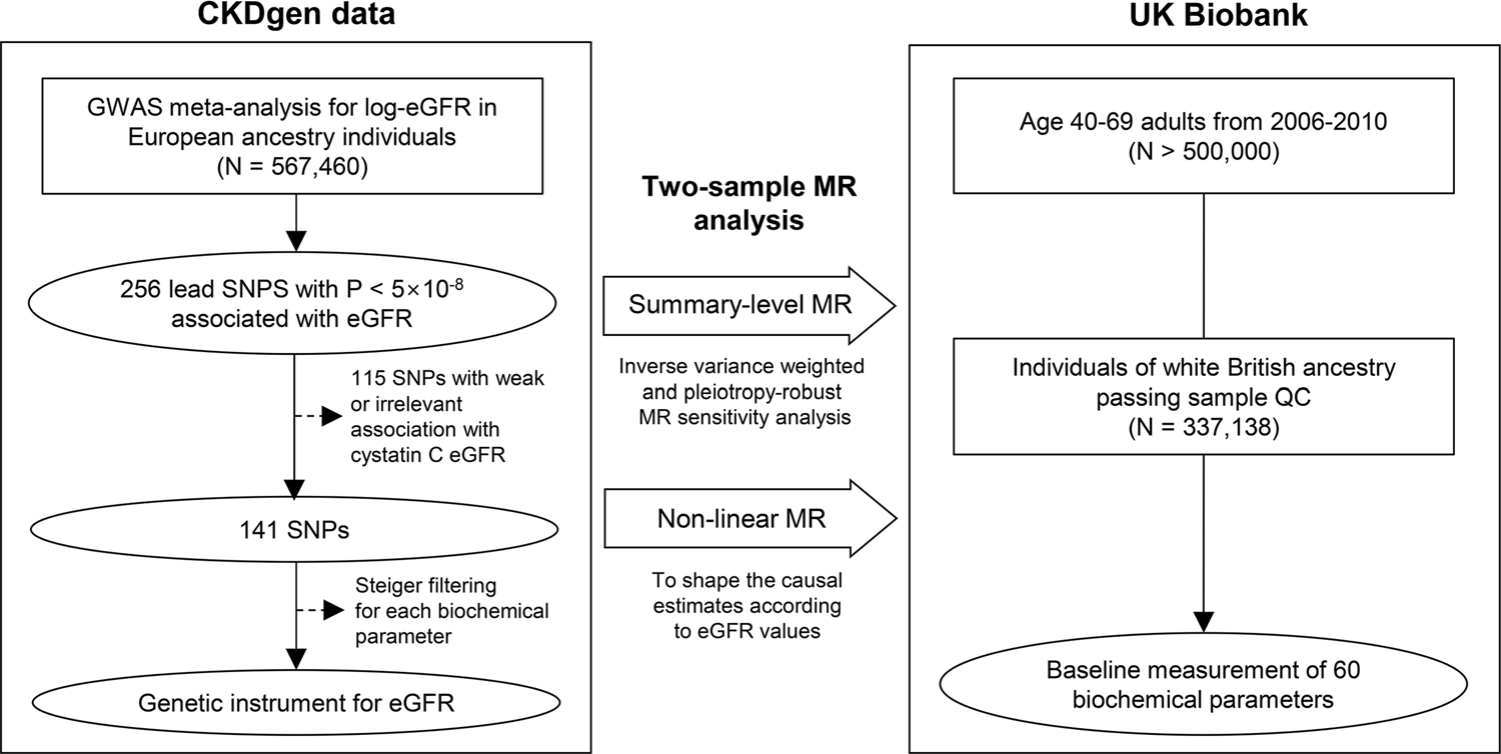
Study flow diagram. In this Mendelian randomization (MR) study, genetic instruments for eGFR were developed from the CKDGen data of European ancestry (*N* = 567,460). Two-sample MR analysis was performed on 60 biochemical parameters measured in 337,138 white UK Biobank participants of British ancestry. A causal estimate significant by the inverse variance–weighted, MR-Egger, and weighted-median methods was considered to indicate the presence of a causal effect. Additional nonlinear MR analysis was performed to investigate the shapes of the causal estimates according to ranges of eGFR values.

The CKDGen data provided the genetic instruments and outcome biochemical parameters were identified in the UK Biobank dataset (Fig. 1). The baseline characteristics of the 337138 white British UK Biobank participants with genotype information are presented in Supplementary Table 1. The study population had a median age of 58 years old and 46.3% had male sex. The median eGFR value was 92.5 mL/min/1.73 m^2^, with a 4.8% prevalence of diabetes and 20.9% a history of hypertension medication.

### Causal estimates of the effects of eGFR on biochemical parameters

**Fig. 2:**
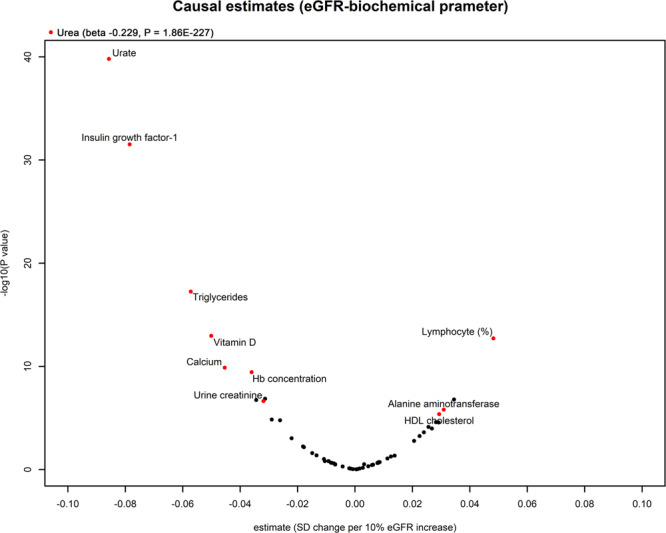
Summary plot demonstrating the estimate effect sizes and significance level (*P*-values). The *X*-axis indicates the causal estimate effect sizes (by the fixed-effects inverse variance–weighted method), which were normalized to a 10% eGFR increase per standard deviation change in a biochemical parameter. The *Y*-axis indicates the -log_10_(*P*-value) to demonstrate a biochemical with a lower *P*-value to be presented in the upper part. The names of the biochemical parameters with significant causal estimates toward both by inverse variance–weighted, MR-Egger, and weighted-median methods are presented. The dots for the consistently significant findings are presented as red. As the causal estimates toward urea, which was the most prominent significant finding, had very low *P*-value, the parameter is separately marked in the left-upper corner of the figure.

We instrumented 140 SNPs that were in genome-wide significant association with log-transformed eGFR values based on creatinine levels in the CKDGen data and also with consistent association with cystatin C-based eGFR values in the UK Biobank data (Supplementary Data 1). By summary-level MR analysis, We found total of 11 biochemical parameters that showed consistently significant association (*P* < 0.05/60) with genetically predicted eGFR values also supported by significant (*P* < 0.05) pleiotropy-robust MR results (Table 1 and Fig. 2). Genetically predicted higher eGFR was significantly associated with lower values of hemoglobin concentration, urine creatinine concentration, serum triglycerides, insulin growth-factor 1, urate, urea, and vitamin D. On the other hand, genetically predicted higher eGFR value was significantly associated with higher levels of lymphocyte percentage, serum alanine aminotransferase, and high-density lipoprotein cholesterol. All of the above significant results were supported by pleiotropy-robust MR sensitivity analysis, including MR-Egger and the weighted-median method. The *P*-value of the intercept in MR-Egger indicated the absence of significant directional pleiotropic effects in the above findings. The leave-one-out analysis results (Supplementary Figs. 1–11) demonstrated an absence of a notable outlier-effect in the calculated causal estimates.

**Table 1:** Significant causal estimates of 11 biochemical parameters from genetically predicted eGFR.

Outcome	N of instrumented SNPs after Steiger filtering	MR-Egger intercept P	MR methods	beta	standard error	P
**Blood cell parameters**						
Hb concentration	115	0.450	FE-IVW	−0.0360	0.0057	3.67E-10
			RE-IVW	−0.0360	0.0173	3.71E-02
			Weighted median	−0.0352	0.0142	1.31E-02
			MR Egger	−0.0936	0.0182	0.09E-03
Lymphocyte (%)	131	0.243	FE-IVW	0.0482	0.0066	1.94E-13
			RE-IVW	0.0482	0.0121	6.40E-05
			Weighted median	0.0359	0.0116	1.99E-03
			MR Egger	0.0503	0.0175	1.00E-03
**Biochemistry assays**						
Urine creatinine	139	0.260	FE-IVW	−0.0319	0.0062	2.30E-07
			RE-IVW	−0.0319	0.0102	1.86E-03
			Weighted median	−0.0240	0.0113	3.32E-02
			MR Egger	−0.0328	0.0155	1.60E-02
Alanine aminotransferase	132	0.399	FE-IVW	0.0309	0.0064	1.58E-06
			RE-IVW	0.0309	0.0115	7.09E-03
			Weighted median	0.0628	0.0119	1.47E-07
			MR Egger	0.0691	0.0175	1.00E-03
Urea	108	0.597	FE-IVW	−0.2293	0.0071	1.86E-227
			RE-IVW	−0.2293	0.0181	6.30E-37
			Weighted median	−0.2351	0.0159	1.50E-49
			MR Egger	−0.2774	0.0235	1.00E-03
Calcium	129	0.484	FE-IVW	−0.0454	0.0071	1.34E-10
			RE-IVW	−0.0454	0.0173	8.81E-03
			Weighted median	−0.0496	0.0168	3.06E-03
			MR Egger	−0.0342	0.0213	4.90E-02
HDL cholesterol	130	0.438	FE-IVW	0.0293	0.0064	4.23E-06
			RE-IVW	0.0293	0.0132	2.67E-02
			Weighted median	0.0403	0.0121	8.48E-04
			MR Egger	0.0485	0.0169	2.00E-03
Triglycerides	127	0.453	FE-IVW	−0.0572	0.0066	5.65E-18
			RE-IVW	−0.0572	0.0127	6.68E-06
			Weighted median	−0.0608	0.0118	2.30E-07
			MR Egger	−0.1070	0.0180	1.00E-03
Insulin-like growth factor-1	124	0.638	FE-IVW	−0.0785	0.0066	3.02E-32
			RE-IVW	−0.0785	0.0125	3.10E-10
			Weighted median	−0.0729	0.0126	7.55E-09
			MR Egger	−0.0788	0.0179	1.00E-03
Urate	103	0.161	FE-IVW	−0.0857	0.0064	1.59E-40
			RE-IVW	−0.0857	0.0220	9.63E-05
			Weighted median	−0.0983	0.0167	4.06E-09
			MR Egger	−0.0749	0.0205	1.00E-03
Vitamin D	137	0.110	FE-IVW	−0.0501	0.0067	1.10E-13
			RE-IVW	−0.0501	0.0112	7.79E-06
			Weighted median	−0.0420	0.0120	4.79E-04
			MR Egger	−0.0850	0.0188	1.00E-03

*SNP* single nucleotide polymorphism, *MR* Mendelian randomization, *FE* fixed-effects, *RE* random-effects, *IVW* inverse variance–weighted.

The numbers of SNPs instrumented to genetically predict eGFR were determined after Steiger filtering for each outcome to exclude the variants with reverse causal effects.

The unit of the causal estimates was from a 10% increase in genetically predicted eGFR on a standard deviation change in a biochemical parameter.

The statistical significance was defined as Bonferroni-adjusted significant (*P* < 0.05/60) by the fixed-effects inverse variance weighted method and nominally significant (*P* < 0.05) results by other pleiotropy-robust MR methods.

Regarding other variables (Supplementary Data 2), the fixed-effects inverse variance–weighted model provided significant (*P* < 0.05/60) causal estimates of red blood cell counts, hemoglobin proportion, platelet count, plateletcrit, mean platelet volume, lymphocyte count, neutrophil proportion, eosinophil proportion, mean sphered cell volume, serum albumin level, and aspartate aminotransferase levels. There were some suggestive findings supported by pleiotropy-robust MR analysis, towards plateletcrit, mean platelet volume, neutrophil proportion, mean sphered cell volume, and serum albumin, as the causal estimates were mostly significant by random-effects inverse variance weighted method and weighted-median methods. MR-Egger regression frequently provided attenuated causal estimates, but the directions of the estimates were similar to the main causal estimates and MR-Egger intercept *P*-value indicated the absence of a directional pleiotropy, which is supportive of the main causal estimates.

### Nonlinear MR analysis

**Fig. 3:**
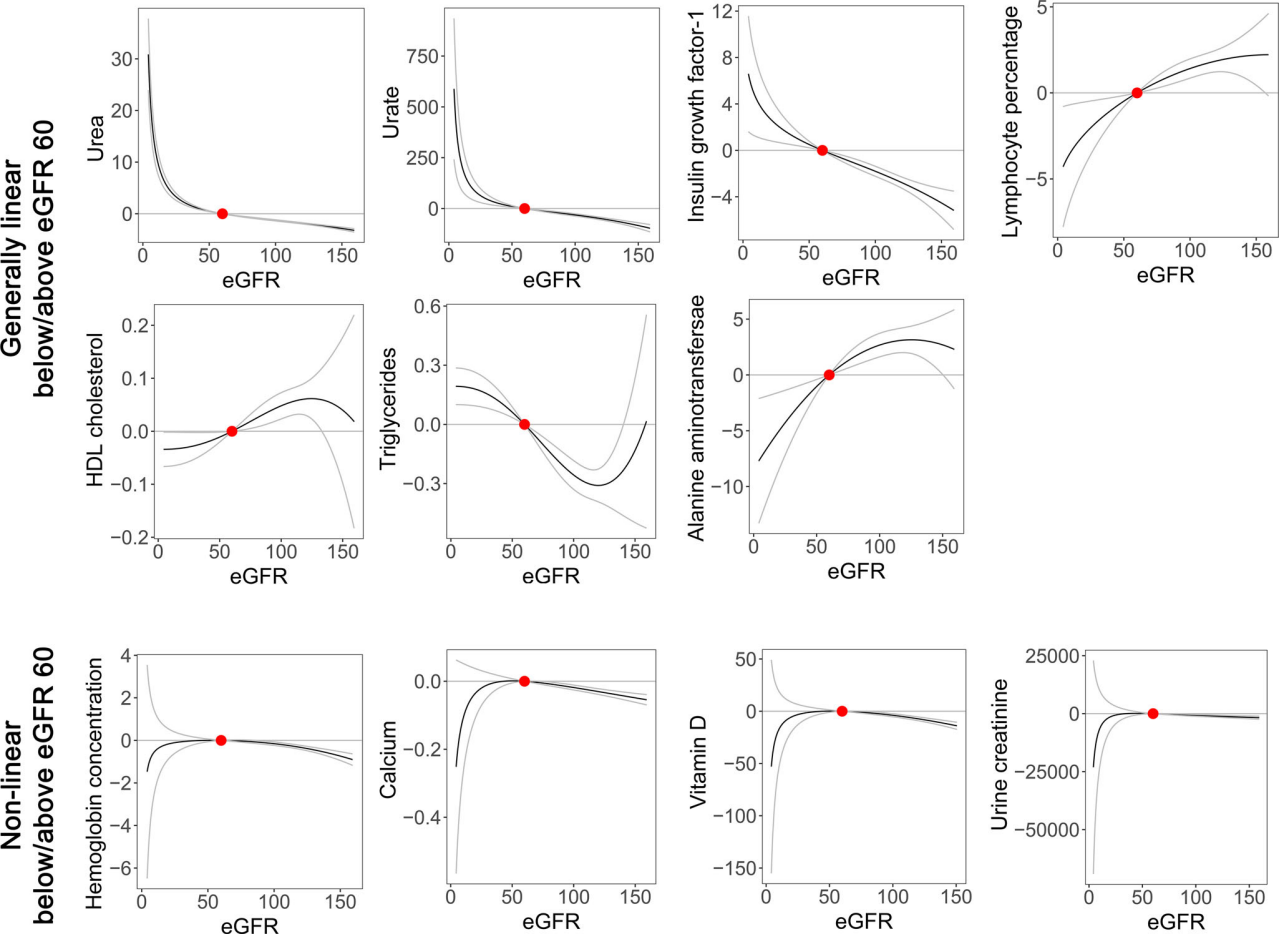
Nonlinear Mendelian randomization analysis plots. The fractional polynomial method of degree 2 was implemented to illustrate the shapes of causal estimates according to eGFR values. The eGFR values were calculated based on the creatinine–cystatin C CKD-EPI equation in the UK Biobank (available in 321,024 individuals). Age, sex, and 10 genetic principal components were included as adjusted covariates. The reference points (red dots) were set to eGFR 60 mL/min/1.73 m^2^, which is a conventional threshold to indicate reduced kidney function. The biochemical parameters for which the causal estimates were consistently significant in the summary-level MR were assessed by nonlinear MR analysis. The gray lines indicate the 95% confidence interval. The unit of eGFR was mL/min/1.73 m^2^, and the unit of measurements for biochemical parameters were as follows: hemoglobin concentration (grams/deciliter), lymphocyte percentage (percent), urine creatinine (micromole/L), alanine aminotransferase (U/L), urea (mmol/L), calcium (mmol/L), HDL cholesterol (mmol/L), insulin-like growth factor-1 (mmol/L), triglycerides (mmol/L), urate (µmol/L), and vitamin D (nmol/L).

The 11 consistently significant signals were further subjected to non-linear MR analysis to investigate the shape of the relation (Fig. 3).

The non-linear MR analysis results towards urea, urate, insulin growth factor-1, lymphocyte percentage, HDL cholesterol, triglycerides, and alanine aminotransferase were considered generally linear with the same direction as the main causal estimates in below and above eGFR 60 mL/min/1.73 m^2^. For urea and urate outcomes, the slope was even steep in those with eGFR < 60 mL/min/1.73 m^2^, and lower genetically predicted eGFR was associated with exponentially higher urea and urate values.

On the other hand, the causal estimates were in different directions (e.g. non-linear) in below and above eGFR 60 mL/min/1.73 m^2^ towards hemoglobin concentration, calcium, vitamin D, and urine creatinine outcomes. The main causal estimates were in the same direction of shape as the findings in eGFR ranges > 60 mL/min/1.73 m^2^, however, in the below ranges, the shape was rather in the opposite direction with large confidence intervals.

### Observational associations

**Fig. 4:**
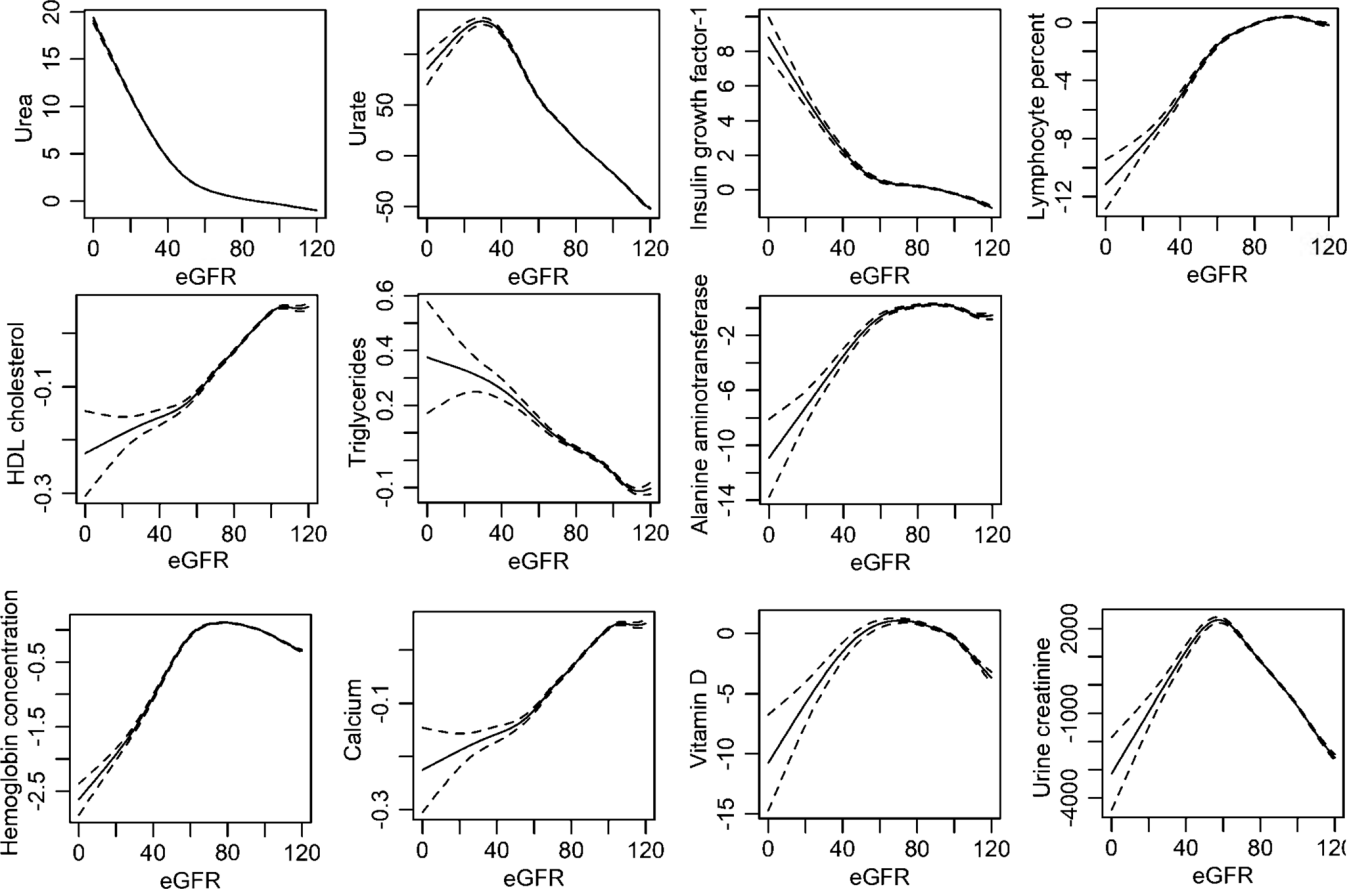
Cross-sectional, observational associations between eGFR and biochemical parameters. The generalized additive model adjusted for age, sex, and body mass index was used to plot the shapes of the observational associations. The eGFR values were calculated based on the creatinine–cystatin C CKD-EPI equation in the UK Biobank (available in 321,024 individuals). The *Y*-axes indicate the values predicted by the generalized additive model. The biochemical parameters for which the causal estimates were consistently significant in the summary-level MR were assessed by cross-sectional observational analysis. The dashed-lines indicate the 95% confidence intervals. The unit of eGFR was mL/min/1.73 m^2^, and the unit of measurements for biochemical parameters were as follows: hemoglobin concentration (grams/deciliter), lymphocyte percentage (percent), urine creatinine (micromole/L), alanine aminotransferase (U/L), urea (mmol/L), calcium (mmol/L), HDL cholesterol (mmol/L), insulin-like growth factor-1 (mmol/L), triglycerides (mmol/L), urate (µmol/L), and vitamin D (nmol/L).

We further investigated the observational association between eGFR values and 11 biochemicals that showed significant findings in the MR analysis (Fig. 4).

Towards biochemical parameters that showed a generally linear association with genetically predicted eGFR values in MR analysis showed similar observational relations. Higher eGFR values were prominently associated with lower urea, urate, insulin growth factor-1, and triglyceride values. On the other hand, higher eGFR values were associated with higher lymphocyte percentage, HDL cholesterol, and alanine aminotransferase.

Regarding biochemical parameters that showed a generally non-linear association with genetically predicted eGFR in MR analysis, the shape of the observational associations were heterogeneous. Higher eGFR values were associated with higher calcium levels. Otherwise, inverse U-shaped association was shown between hemoglobin concentration, vitamin D, and urine creatinine levels.

## Discussion

In this MR analysis, we identified that genetically predicted eGFR values were significantly associated with various biochemical parameters and that the cause–effect curves may have diverse shapes, identified from non-linear MR. With our efforts to attain the MR assumptions by performing pleiotropy-robust MR analysis, this study supports the causal effects of eGFR on diverse biochemical parameters

The consistently significant findings by our MR analysis are explained below. Clinically relevant findings were identified for blood urea, and urate levels, as the causal effects were prominent in the eGFR range of <60 mL/min/1.73 m^2^. Blood urea is a secondary kidney function parameter, and serum albumin might have been affected by impaired volume homeostasis or urine albumin loss related to kidney function impairment. Urate is a well-known parameter that is closely associated with eGFR values^3,11^, particularly in CKD patients, and our results showed that eGFR decline would causally increase serum urate levels.

Lymphopenia is a frequently reported condition in patients with impaired kidney function^12,13^, and our MR results suggest that the lymphocyte percentage starts to decrease in the early stage of eGFR reduction. In addition, this may support the clinical importance of the neutrophil-dominant state in CKD patients and the impairment of adaptive immunity in those with reduced eGFR^14,15^.

A higher genetically predicted eGFR was generally associated with higher HDL cholesterol and lower triglyceride levels. Along with previous MR findings suggesting that HDL cholesterol causally affects kidney function^16^, an additional reverse-direction effect was identified herein, indicating that a decline in kidney function may causally aggravate cholesterol profiles. This would provide an additional explanation for the close linkage between CKD and cardiovascular outcomes, with dyslipidemia possibly being involved in both comorbidities.

Several previous studies reported that certain serum aminotransferase levels were reduced in patients with impaired kidney function^17,18^. Although the mechanism remains uncertain, our MR results support the idea that a decrease in eGFR may cause lower alanine aminotransferase levels. Previous studies have suggested that low kidney function may inhibit hepatic release or conversion of aminotransferase related to pyridoxine deficiency or hyperhomocysteinemia^19,20^. As we identified the observed association as similar to the shape of actual causal effects from eGFR, additional study is warranted to investigate the mechanism of the finding.

We found that a higher eGFR might causally decrease insulin-like growth factor-1. A similar observational association was also reported previously in approximately 2000 men in the Study of Health in Pomerania cohort and over 5000 participants in the Third National Health and Nutrition Examination Survey^21,22^. The clinical importance of the growth hormone/insulin-like growth factor-1 axis regarding kidney function has been noted previously, and insulin-like growth factor-1 is considered protective against kidney injury^23^. Resistance to insulin-like growth factor-1 has been reported in CKD patients; thus, a decrease in eGFR may causally elevate insulin-like growth factor-1 because of the lack of sensitivity. More robust evidence including the measurement of growth hormone or insulin-like growth factor–binding protein levels should be pursued to reveal the causal effects of kidney function decline on the growth hormone axis.

Finally, there were additional findings that should be interpreted carefully because the causal effects reported by the summary-level MR generally reflected the causal estimates in supranormal ranges, while the nonlinear MR demonstrated non-linear exposure–outcome relationships. The decreased levels of hemoglobin and calcium in people with increased genetically predicted eGFR may stem from the causal effects of kidney hyperfiltration^24^, which is another impaired kidney function state. Given the reversed direction when eGFR < 60 mL/min/1.73 m^2^ as well as the findings from the observational investigations, the existing knowledge that kidney function decline can cause calcium imbalance or anemia should be heeded than the current causal estimates^25,26^. This similarly applies to the causal estimates for vitamin D levels, as kidney hyperfiltration may be causally linked to vitamin D deficiency^4^. The effects of severe eGFR decline were not apparent in this MR analysis, but previous clinical evidence clearly suggests that CKD with eGFR < 60 mL/min/1.73 is associated with vitamin D deficiency.

There are some limitations to this study. First, there were some U-shaped associations between genetically predicted eGFR and biochemical parameters, although the summary-level MR analysis assumes that the exposure–outcome relationship is linear. It should be noted that some causal estimates did not reflect the findings in certain eGFR ranges, particularly eGFR < 60 mL/min/1.73 m^2^. In some parameters, observational associations suggested that conventional knowledge (e.g., chronic kidney disease causing anemia) would hold, but significant causal estimates for eGFR < 60 mL/min/1.73 m^2^ were not revealed in the current MR results. As the UK Biobank data are affected by healthy volunteer bias, the causal estimates may be subject to selection bias due to the underrepresentation of severe kidney function decline in the sample. Second, as a false-negative finding is possible in two-sample MR, modest causal effects may also be present for other parameters that were not revealed to be affected by genetically predicted eGFR in the current analysis. Therefore, one should be cautious about concluding a null causal effect based on the negative findings of the current study. Third, the reverse-directional causal effect was not assessed herein, thus, one should not consider the possibility of such effect from biochemical parameters on eGFR values based on some negative results. Fourth, the analysis was restricted to individuals of European ancestry; thus, generalizability is not confirmed for other ethnic populations. Fifth, the current MR analysis could not confirm a mechanistic explanation for the suggested causal effects. Lastly, the inherent limitation of MR analysis that the validity is based on some non-testable assumption should be reminded, and a conclusion of the causal effects should be made based on additional evidence.

In conclusion, eGFR causally affects various biochemical parameters in diverse patterns. Clinicians may consider the suggested causal effects from eGFR on laboratory findings in order to appropriately interpret observed associations between kidney function and biochemical measurements. Future studies are warranted to investigate the mechanism and expand the clinical utility of the causal effects of kidney function on biochemical parameters.

## Methods

### Ethical considerations

The study was approved by the institutional review boards of Seoul National University Hospital (No. E-2012-004-1177) and the UK Biobank consortium (application No. 53799). The study was performed in accordance with the Declaration of Helsinki. The requirement for informed consent was waived because the study analyzed public databases.

### Genetic instruments for kidney function

We used the CKDGen phase 4 GWAS meta-analysis results (URL: https://ckdgen.imbi.uni-freiburg.de/), including 567,460 European ancestry individuals to develop genetic instruments for kidney function (Fig. 1)^27,28^. The 256 single nucleotide polymorphisms (SNPs) with genome-wide significant association with log-transformed creatinine-based eGFR were screened and 141 SNPs which also showed relevant association cystatin C-based eGFR were used as the instruments, following our previous studies^8–10^. We used Steiger filtering to include only variants the direction of effects were from eGFR towards biochemical parameters to exclude reverse-directional effect. Steiger filtering calculates the explained variance towards exposure and outcome phenotype, respectively, and extracts the SNPs with larger explained variance towards exposure, ensuring the direction of the genetic effect. We found that without the Steiger filtering process, the causal estimates are substantially biased by few SNPs that reflects the reverse-directional effect, namely, a biochemical parameter effect on eGFR (Supplementary Table 2 and Supplemetary Fig. 12). Total of 140 SNPs were screened as one SNP was unidentifiable in the UK Biobank data and the SNPs passing the Steiger filtering process were used in the according analyses. The detailed information of the genetic instruments is provided in Supplementary Data 1.

### Outcome biochemical measurements in the UK Biobank data

The UK Biobank is a population-scale prospective cohort that recruited > 500,000 participants aged 40–69 years from diverse regions in 2006–2010 (Fig. 1)^29^ (UK Biobank data, UK Biobank consotium (URL: https://www.ukbiobank.ac.uk/)). The database includes various clinicodemographic information and deep genotyping data and thus has been widely used for genetic studies, including MR analysis. We collected the information from 337,318 white British ancestry samples in the UK Biobank data.

We collected 60 biochemical parameters available in the UK Biobank as the outcome data. The laboratory information measured at baseline visits included diverse complete blood counts and proportions, liver enzymes, lipid profiles, electrolyte levels, urate values, glycemia-related information (including hemoglobin A1c), total proteins or albumin, sex-hormone or sex hormone–binding globulin values, and insulin-like growth factor 1 (IGF-1). We also collected urine electrolyte or creatinine parameters. The biochemical parameters were scaled to standard deviation units to give comparable results between the phenotypes, and summary statistics for the instrumental SNPs and the outcome traits were generated by the GWAS by linear regression model, adjusted for age, sex, and 10 genetic principal components. The GWAS summary statistics towards the biochemical parameters are provided in Supplementary Data 3.

### Data collection

The information of the data from the UK Biobank consortium are available online (URL: https://www.ukbiobank.ac.uk/data-showcase/), and the information is identified by field IDs.

Age (Field ID 21003) and sex (Field ID 31) information was collected. Baseline eGFR values were calculated from the information of serum creatinine levels (Field ID 30700) and cystatin C levels (Field ID 10720), along with information of ethnicity (Field ID 21000), calculated by the CKD-EPI equation.

For genotype data, UK Biobank provides standardized imputed genotype data that can be used for various forms of researches. The details of the genotype data provided by the consortium can be found in a reference paper^29^, along with the details regarding the imputation (URL: https://biobank.ndph.ox.ac.uk/showcase/ukb/docs/impute_ukb_v1.pdf).

The UK Biobank blood assay results, available in the time of this study, include blood count (category ID 100081), blood biochemistry (category ID 17518), and infectious disease antigen assay results (category ID 51428). Among them, we included 60 available biochemistry results from blood count results and blood biochemistry data. All blood samples were collected at the timing of baseline visits (2006–2010). The blood cell counts (category ID 100081) were obtained by Beckman Coulter LH750 Hematology Analyzer from samples collected in from 4 mL EDTA vacutainers. The serum biochemistry results (category ID 17518) were obtained from 10 immunoassay analyzers (6x DiaSorin Liaison XL & 4x Beckman Coulter DXI 800) and 4 clinical chemistry analyzers (2x Beckman Coulter AU5800 & 2x Siemens Advia 1800).

The other details regarding the quality control metrices and assay methods are presented in the companion document provided by the UK Biobank consortium; Blood cell count (URL: https://biobank.ndph.ox.ac.uk/ukb/ukb/docs/haematology.pdf), and serum biochemistry (URL: httpls://biobank.ndph.ox.ac.uk/ukb/ukb/docs/serum_biochemistry.pdf).

The numbers of missing cases for a biochemical assay result, among the 337,138 white British ancestry samples included in the genetic analysis, are as below: white blood cell count (*N* = 9979), red blood cell count (*N* = 9975), hemoglobin concentration (*N* = 9976), hematocrit (*N* = 9975), mean corpuscular volume (*N* = 9977), mean corpuscular hemoglobin (*N* = 9978), mean corpuscular hemoglobin concentration (*N* = 9982), red blood cell distribution width (*N* = 9977), platelet count (*N* = 9976), plateletcrit (*N* = 9979), mean platelet volume (*N* = 9980), platelet distribution width (*N* = 9980), lymphocyte count (*N* = 10,542), monocyte count (*N* = 10,542), neutrophil count (*N* = 10,542), eosinophil count (*N* = 10,542), basophil count (*N* = 10,542), nucleated red blood cell count (*N* = 10,550), lymphocyte percentage (*N* = 10,538), monocyte percentage (*N* = 10,538), neutrophil percentage (*N* = 10,538), eosinophil percentage (*N* = 10,538), basophil percentage (*N* = 10,538), nucleated red blood cell percentage (*N* = 10,553), reticulocyte percentage (*N* = 15,273), reticulocyte count (*N* = 15,272), mean reticulocyte volume (*N* = 15,272), mean sphered cell volume (*N* = 15,272), immature reticulocyte fraction (*N* = 15,272), high light scatter reticulocyte percentage (*N* = 15,272), high light scatter reticulocyte count (*N* = 15,272), urine creatinine (*N* = 9673), urine potassium (*N* = 10,381), urine sodium (*N* = 10,367), serum albumin (*N* = 42,776), alkaline phosphatase (*N* = 15,699), alanine aminotransferase (*N* = 15,838), apolipoprotein A (*N* = 44,521), apolipoprotein B (*N* = 17,271), aspartate aminotransferase (*N* = 16,903), direct bilirubin (*N* = 63,663), urea (*N* = 15,924), calcium (*N* = 42,884), total cholesterol (*N* = 15,713), C-reactive protein (*N* = 16,410), gamma glutamyl transferase (*N* = 15,872), glucose (*N* = 43,087), glycated hemoglobin A1c (*N* = 15,843), HDL cholesterol (*N* = 42,902), insulin growth factor-1 (*N* = 17,439), LDL cholesterol (*N* = 16,319), lipoprotein A (*N* = 81,569), phosphate (*N* = 43,337), sex hormone binding globulin (*N* = 45,606), total bilirubin (*N* = 17,058), testosterone (*N* = 45,700), total protein (*N* = 43,102), triglycerides (*N* = 15,977), urate (*N* = 16,127), vitamin D (*N* = 29,845).

The details of each biochemical parameter, number of observations, and basic characteristics of distribution can be found in the UK Biobank data showcase website (URL: https://www.ukbiobank.ac.uk/data-showcase/).

### MR assumptions

We made efforts to confirm the fulfillment of the three key MR assumptions that should be fulfilled to enable causal inference^5^. First, we addressed the relevance assumption, i.e., that the genetic instruments are strongly associated with the exposure of interest. As the instruments were the SNPs with genome-wide significant associations with log(eGFR) values, this assumption was considered to be fulfilled. The second and third assumptions indicate the absence of a horizontal pleiotropic pathway. The independence assumption is that genetic instruments should not be associated with a confounder phenotype. The exclusion-restriction assumption is that the genetic effects should occur through the exposure of interest. Although identifying every possible confounder phenotype is difficult and a statistical confirmation of the exclusion-restriction assumption is impossible, there are pleiotropy-robust MR analysis methods, usually performed as sensitivity analyses in MR studies, which partially relax the second and third assumptions^30^. We performed representative pleiotropy-robust MR analyses to yield causal estimates that sufficiently attained the assumptions. In addition, we paid attention to the direction of the variants’ effects, as a direct effect of the SNPs on outcome traits rather than on kidney function would violate the exclusion-restriction assumption.

### Summary-level MR analysis

We first applied the conventional fixed-effects inverse variance–weighted method as the main MR analysis. However, as the fixed-effects model can be biased by a variant-specific effect and be over-precise, we also applied a random-effects model, which can address the issue of heterogeneity, allowing a balanced pleiotropic effect. As a pleiotropy-robust MR sensitivity analysis, we performed an MR-Egger regression analysis with bootstrapped error^31^. Additional weighted-median method as performed which yields valid causal estimates relaxing the MR assumptions in upto 50% of genetic instruments^32^. We considered a Bonferroni-adjusted significant result (*P* < 0.05/60) in the fixed-effects inverse variance–weighted model, supported by the results from random-effects inverse variance–weighted model, MR-Egger regression with bootstrapped error, and median-based methods with conventional significance threshold (*P* < 0.05), to be a significant finding. The significant findings were inspected by leave-one-out analysis to investigate whether an extreme outlier suspected with pleiotropic effect biased the findings. The summary-level MR analysis was performed using the “TwoSampleMR” package in R (version 0.4.26)^33^. All causal estimates were normalized to units of a 10% change in the eGFR for a 1-standard-deviation change in an outcome.

### Non-linear MR analysis

As summary-level MR assesses the linear association between a genetic predisposition for an exposure and outcome, additional non-linear MR analysis is necessary to understand the shape of the assessed cause–effect relationship^34^. As supranormal eGFR value (“kidney hyperfiltration”) is considered another state of kidney dysfunction^35,36^, we used the non-linear MR analysis to additionally demonstrate the shape of the linkage between genetically predicted eGFR and biochemical parameters. In non-linear MR, study population is stratified based on instrument-free exposure, the residual variation which reflect the non-genetic portion of eGFR. This is because directly dividing the population by eGFR phenotype would invalidate the MR assumption causing collider bias. Next, the causal estimates between genetically predicted eGFR and outcome are calculated in each strata (e.g. localized averaged causal estimates), and the estimates are meta-regressed to demonstrate exposure–outcome relationship.

Among the 337,138 white British-descended UK Biobank participants, 321,024 individuals with available baseline eGFR values calculated by the creatinine–cystatin C CKD-EPI method, measured in the same biospecimens as the outcome biochemical parameters, were included in the analysis^8^. We calculated allele scores for eGFR by the genetic instruments after Steiger filtering with PLINK 2.0 and used the scores as the exposure variables in each analysis for a given outcome. We implemented the fractional polynomial method of degree 2, which is more flexible than the degree 1 model, to illustrate the shape of the causal estimates according to eGFR values, setting the reference point to an eGFR of 60 mL/min/1.73 m^2^^34^. Age, sex, and 10 genetic principal components were included as adjusted covariates. The biochemical parameters for which the causal estimates were consistently significant in the summary-level MR were assessed by nonlinear MR analysis. The nonlinear MR analysis was performed with the “nlmr” package in R.^34^

### Observational associations

Along with the causality investigated in the aforementioned MR analysis, we additionally inspected the observational, cross-sectional associations between the biochemical parameters and eGFR in the 337,138 individuals included in the MR analysis. As causal inference is limited with cross-sectional associations, we analyzed the associations for the parameters that showed significant causal estimates in the abovementioned summary-level MR analysis. A generalized additive model adjusted for age, sex, and body mass index was used to plot the shapes of the associations using the “mgcv” package in R.

### Reporting summary

Further information on research design is available in the Nature Research Reporting Summary linked to this article.

## Supplementary information

Supplementary Information

Description of Additional Supplementary Files

Supplementary Data 1

Supplementary Data 2

Supplementary Data 3

Reporting Summary

## Data Availability

The data for this study are available in the public domain. The information towards genetic instruments were provided by the CKDGen data (URL: https://ckdgen.imbi.uni-freiburg.de/) and the results under subheading “Wuttke et al. 2019 publication” was used. The information is freely downloadable in the website, and the Supplementary Data 1 also provides the information. The UK Biobank data can be accessed via the consortium website (https://www.ukbiobank.ac.uk/), under application No 53799. The generated GWAS summary statistics are available in the Supplementary Data 3.
