# Supplementary material for: Mendelian randomization reveals causal effects of kidney function on various biochemical parameters
Source: Commun Biol. 2022 Jul 18;5:713. doi: 10.1038/s42003-022-03659-4 (PMC9293908; doi:10.1038/s42003-022-03659-4)
Supplement: Supplementary file 1 — Supplementary Information [file 42003_2022_3659_MOESM1_ESM.pdf]

Supplementary Material

Causal effects of estimated glomerular filtration rate on various biochemical parameters: A Mendelian randomization study

S Park et al.

Table of contents

Supplementary Table 1. Baseline characteristics and biochemical parameters of the studied white British ancestry individuals of the UK Biobank.

Supplementary Table 2. Causal estimates towards a biochemical parameter, hemoglobin concentration, by MR analysis without Steiger filtering process.

Supplementary Figure 1. Leave-one-out analysis result to inspect the positive findings: hemoglobin concentration.

Supplementary Figure 2. Leave-one-out analysis result to inspect the positive findings: lymphocyte (%).

Supplementary Figure 3. Leave-one-out analysis result to inspect the positive findings: urine creatinine.

Supplementary Figure 4. Leave-one-out analysis result to inspect the positive findings: alanine aminotransferase.

Supplementary Figure 5. Leave-one-out analysis result to inspect the positive findings: urea.

Supplementary Figure 6. Leave-one-out analysis result to inspect the positive findings: calcium.

Supplementary Figure 7. Leave-one-out analysis result to inspect the positive findings: HDL cholesterol.

Supplementary Figure 8. Leave-one-out analysis result to inspect the positive findings: triglycerides.

Supplementary Figure 9. Leave-one-out analysis result to inspect the positive findings: insulin-like growth factor-1.

Supplementary Figure 10. Leave-one-out analysis result to inspect the positive findings: urate.

Supplementary Figure 11. Leave-one-out analysis result to inspect the positive findings: vitamin D.

Supplementary Figure 12. Single-SNP analysis result towards a biochemical parameter, hemoglobin concentration, without Steiger filtering process.

Supplementary Data 1. Genetic instrument for kidney function developed from the GWAS meta-analysis within the individuals of European ancestry of the CKDGen data.

Supplementary Data 2. Non-significant causal estimates of 49 biochemical parameters from genetically predicted eGFR.

Supplementary Data 3. GWAS summary-statistics towards 60 biochemical parameters of 140 SNPs which were instrumented to genetically predict kidney function.

Supplementary Table 1. Baseline characteristics and biochemical parameters of the studied white British ancestry individuals of the UK Biobank.

**Variables**	Number (%) and median [IQR]
**Base characteristics**	
Number of samples	337138
Age (years)	58.0 [51.0;63.0]
Female	181027 (53.7%)
Male	156111 (46.3%)
eGFR (mL/min/1.73 m^2)	92.5 [82.6;99.5]
Body mass index (kg/m^2)	26.7 [24.1;29.8]
Diabetes mellitus	16179 (4.8%)
Hypertension medication	70021 (20.9%)
**Biochemical parameters**	
WBC count (10^9 cells/L)	6.7 [5.6;7.8]
RBC count (10^9 cells/L)	4.5 [4.2;4.8]
Hb (g/dL)	14.2 [13.4;15.1]
Hematocrit (%)	41.1 [38.8;43.5]
Mean corpuscular volume (fL)	91.4 [88.8;94.0]
Mean corpuscular haemoglobin (pg)	31.6 [30.6;32.5]
Mean corpuscular haemoglobin concentration (g/dL)	34.5 [33.9;35.1]
Red blood cell distribution width (%)	13.3 [12.9;13.8]
Platelet count (10^9 cells/L)	248.0 [213.7;287.0]
Platelet crit (%)	0.2 [0.2;0.3]
Mean platelet thrombocyte volume (fL)	9.2 [8.6;9.9]
Platelet distribution width (%)	16.4 [16.1;16.8]
Lymphocyte count (10^9 cells/L)	1.9 [1.5;2.3]
Monocyte count (10^9 cells/L)	0.5 [0.4;0.6]
Neutrophill count (10^9 cells/L)	4.0 [3.3;5.0]
Eosinophill count (10^9 cells/L)	0.1 [0.1;0.2]
Basophill count (10^9 cells/L)	0.0 [0.0;0.0]
Nucleated red blood cell count (10^9 cells/L)	0.0 [0.0;0.0]
Lymphocyte (%)	28.3 [23.7;33.2]
Monocyte (%)	6.9 [5.6;8.3]
Neutrophill (%)	61.4 [55.9;66.6]
Eosinophill (%)	2.1 [1.4;3.2]
Basophill (%)	0.4 [0.3;0.7]
Nucleated red blood cell (%)	0.0 [0.0;0.0]
Reticulocyte (%)	1.3 [1.0;1.6]
Reticulocyte count (10^{12} cells/L)	0.1 [0.0;0.1]
Mean reticulocyte volume (fL)	105.8 [101.4;110.5]
Mean sphered cell volume (fL)	82.7 [79.4;86.1]
Immature reticulocyte fraction (ratio)	0.3 [0.2;0.3]
High light scatter reticulocyte (%)	0.4 [0.2;0.5]
High light scatter reticulocyte count (10^{12} cells/L)	0.0 [0.0;0.0]
Urine creatinine (mmol/L)	7483.0 [4357.0;11956.0]
Urine potassium (mmol/L)	57.3 [37.3;83.5]
Urine sodium (mmol/L)	67.3 [42.4;101.8]
Albumin (g/L)	45.2 [43.5;46.9]
Alkaline phosphatase (U/L)	80.3 [67.3;95.7]
Alanine aminotransferase (U/L)	20.2 [15.4;27.4]
Apolipoprotein A (g/L)	1.5 [1.4;1.7]
Apolipoprotein B (g/L)	1.0 [0.9;1.2]
Aspartate aminotransferase (U/L)	24.4 [21.0;28.8]
Direct bilirubin (umol/L)	1.6 [1.3;2.1]
Urea (mmol/L)	5.3 [4.5;6.2]
Calcium (mmol/L)	2.4 [2.3;2.4]
Cholesterol (mmol/L)	5.7 [4.9;6.4]
C-reactive protein (mg/L)	1.3 [0.7;2.7]
Gamma glutamyl-transferase U/L)	26.2 [18.5;40.9]
Glucose (mmol/L)	4.9 [4.6;5.3]
HbA1c (mmol/mol)	35.1 [32.7;37.7]
HDL cholesterol (mmol/L)	1.4 [1.2;1.7]
IGF-1 (mmol/L)	21.3 [17.6;24.8]
LDL cholesterol (mmol/L)	3.5 [3.0;4.1]
Lipoprotein A (mmol/L)	20.1 [9.3;60.4]
Phosphate (mmol/L)	1.2 [1.1;1.3]
Sex hormone-binding globulin (mmol/L)	45.5 [32.7;64.1]
Total bilirubin (umol/L)	8.1 [6.4;10.4]
Testosterone (nmol/L)	5.2 [1.0;11.7]
Total protein (g/L)	72.2 [69.6;74.9]
Triglycerides (mmol/L)	1.5 [1.1;2.2]
Urate (umol/L)	303.4 [250.8;361.2]
Vitamin D (nmol/L)	48.2 [33.8;63.5]

Supplementary Table 2. Causal estimates towards a biochemical parameter, hemoglobin concentration, by MR analysis without Steiger filtering process.

MR Methods	N of instrumented SNPs	MR-Egger intercept P	beta	Standard error	P
FE-IVW	140	0.450	-0.0789	0.0545	0.1472
RE-IVW			-0.0789	0.2975	0.7908
Weighted median			0.3672	0.1405	0.0129
MR Egger (bootstrap)			0.1341	0.2432	0.284

SNP = single nucleotide polymorphism, MR = Mendelian randomization, FE = fixed-effects, RE = random-effects, IVW = inverse variance–weighted

The numbers of SNPs instrumented to genetically predict eGFR were determined without Steiger filtering, thus, total 140 SNPs available in the UK Biobank were used.

The unit of the causal estimates was from a 10% increase in genetically predicted eGFR on a standard deviation change in a biochemical parameter.

Supplementary Figure 1. Leave-one-out analysis result to inspect the positive findings: hemoglobin concentration.

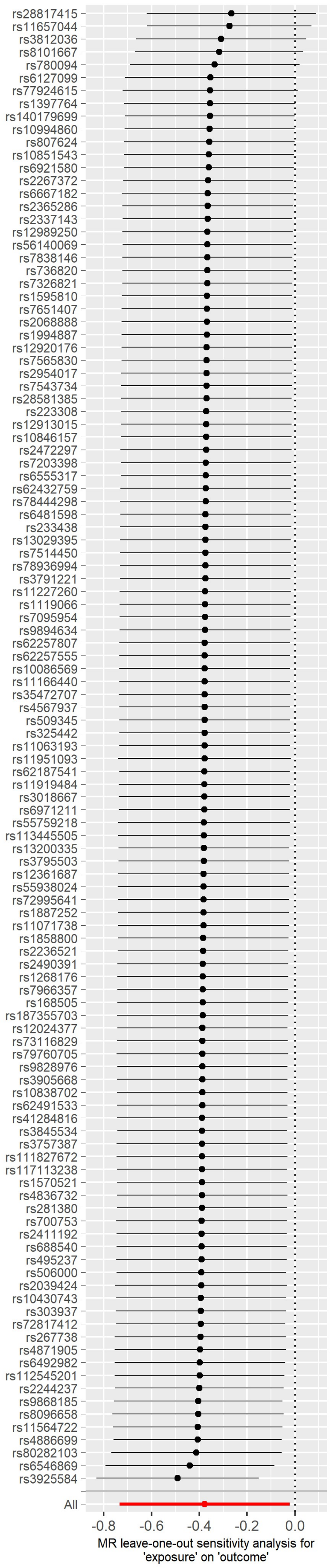

The error bars indicate the 95% confidence intervals.

Supplementary Figure 2. Leave-one-out analysis result to inspect the positive findings: lymphocyte (%).

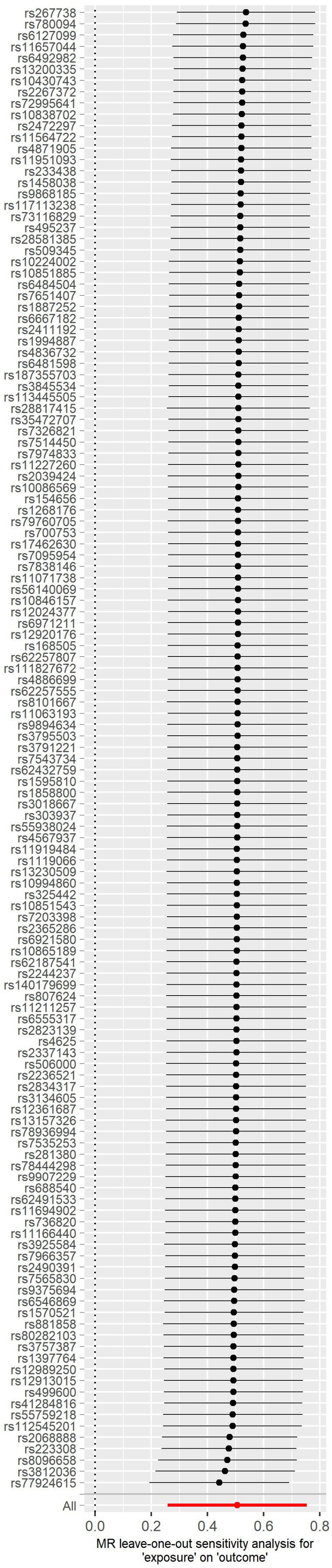

The error bars indicate the 95% confidence intervals.

Supplementary Figure 3. Leave-one-out analysis result to inspect the positive findings: urine creatinine.

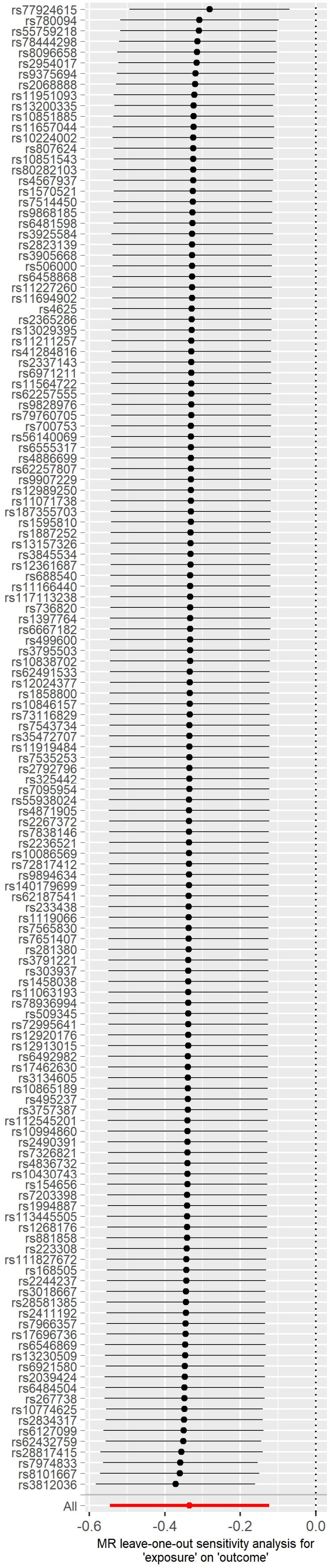

The error bars indicate the 95% confidence intervals.

Supplementary Figure 4. Leave-one-out analysis result to inspect the positive findings: alanine aminotransferase.

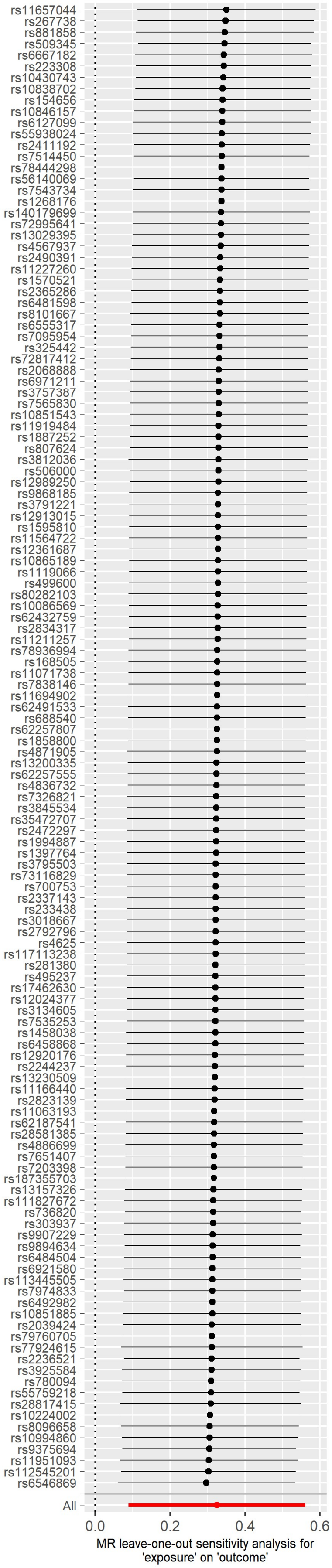

The error bars indicate the 95% confidence intervals.

Supplementary Figure 5. Leave-one-out analysis result to inspect the positive findings: urea.

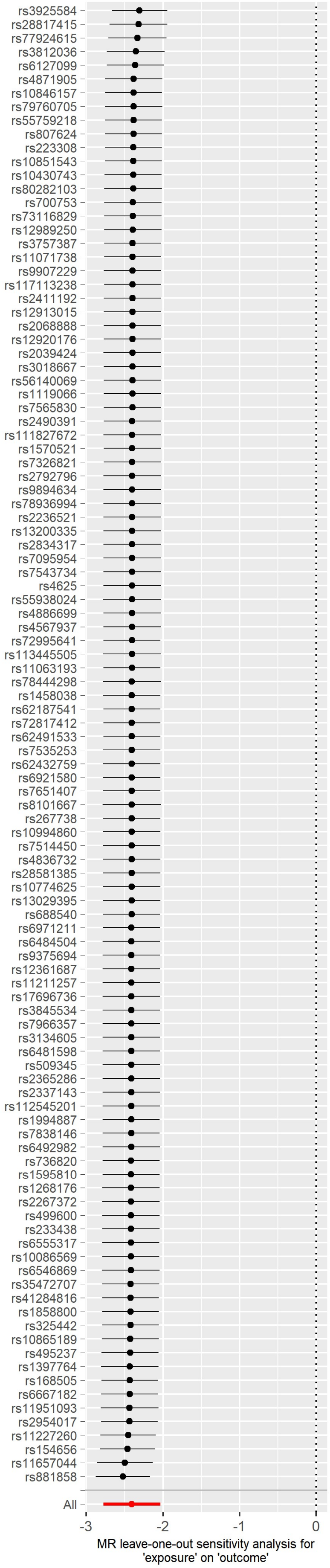

The error bars indicate the 95% confidence intervals.

Supplementary Figure 6. Leave-one-out analysis result to inspect the positive findings: calcium.

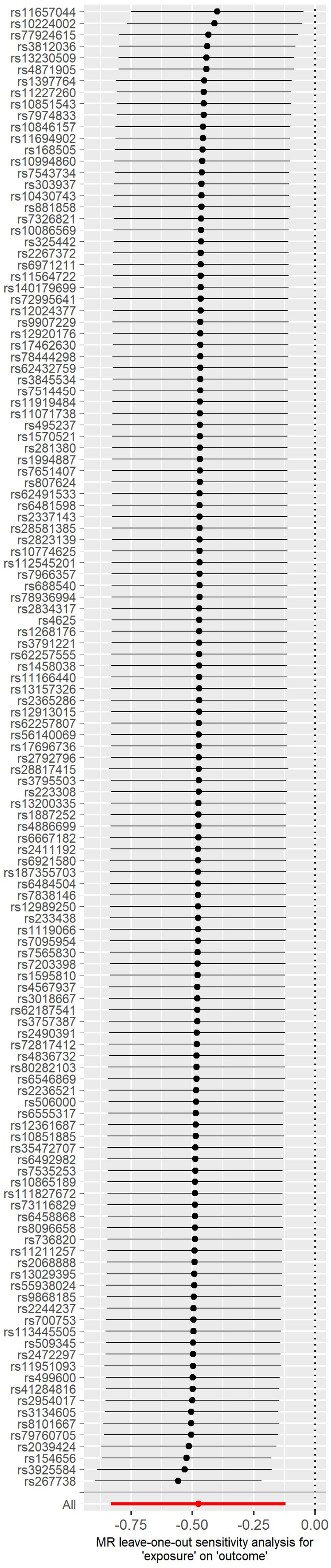

The error bars indicate the 95% confidence intervals.

Supplementary Figure 7. Leave-one-out analysis result to inspect the positive findings: HDL cholesterol.

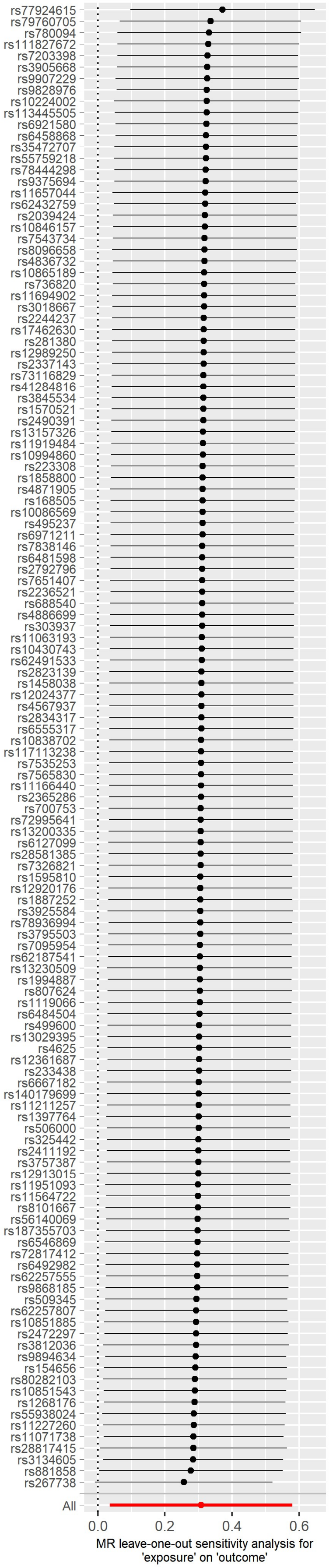

The error bars indicate the 95% confidence intervals.

Supplementary Figure 8. Leave-one-out analysis result to inspect the positive findings: triglycerides.

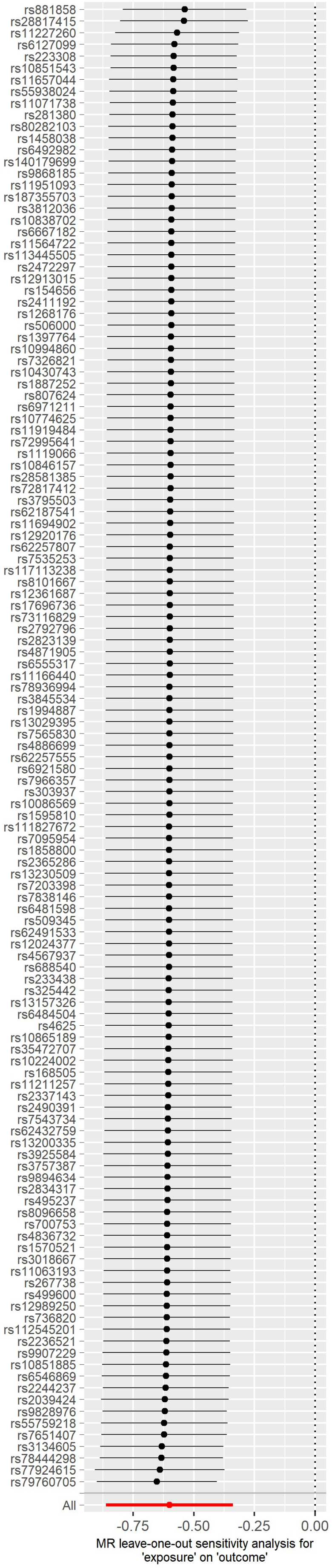

The error bars indicate the 95% confidence intervals.

Supplementary Figure 9. Leave-one-out analysis result to inspect the positive findings: insulin-like growth factor-1.

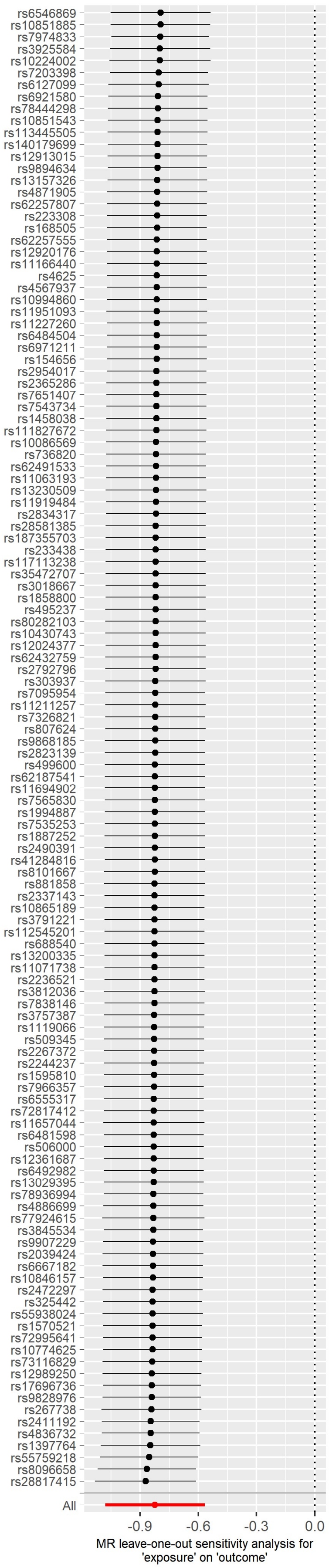

The error bars indicate the 95% confidence intervals.

Supplementary Figure 10. Leave-one-out analysis result to inspect the positive findings: urate.

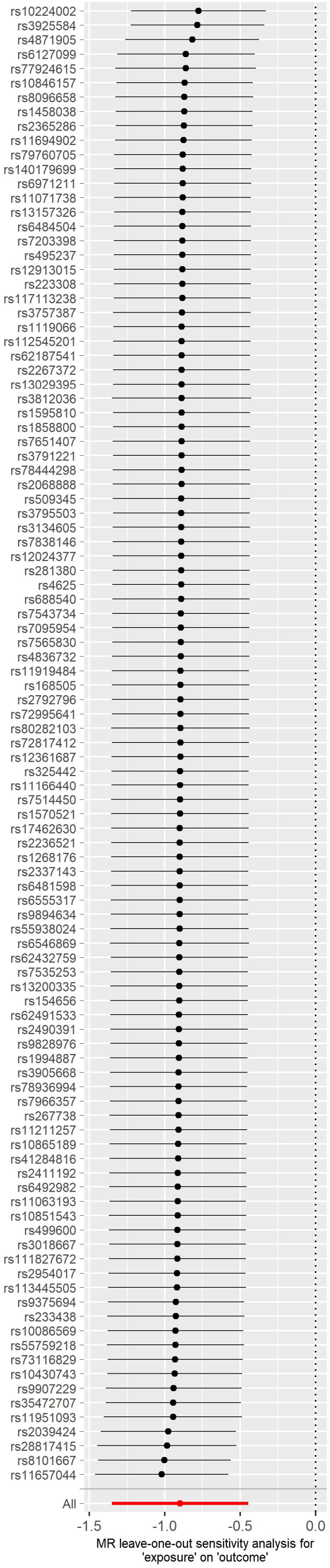

The error bars indicate the 95% confidence intervals.

Supplementary Figure 11. Leave-one-out analysis result to inspect the positive findings: vitamin D.

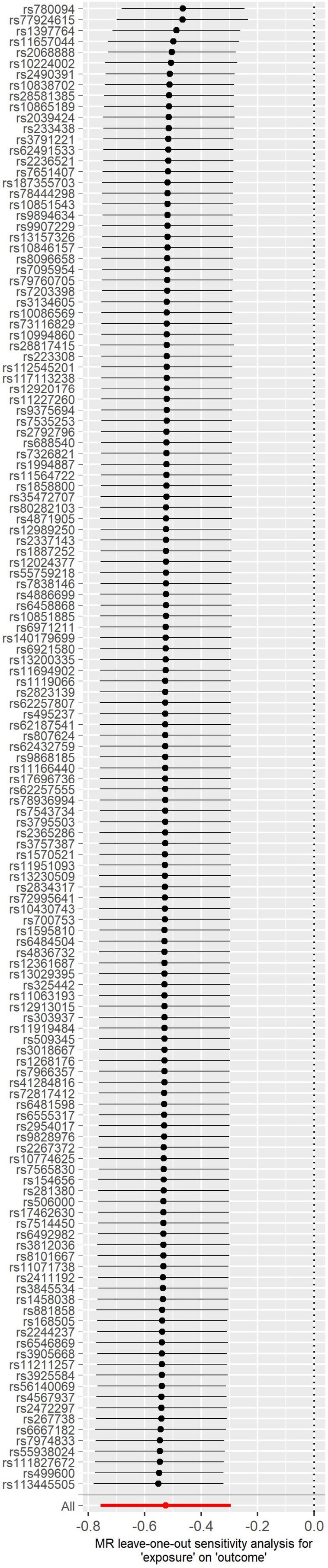

The error bars indicate the 95% confidence intervals.

Supplementary Figure 12. Single-SNP analysis result towards a biochemical parameter, hemoglobin concentration, without Steiger filtering process.

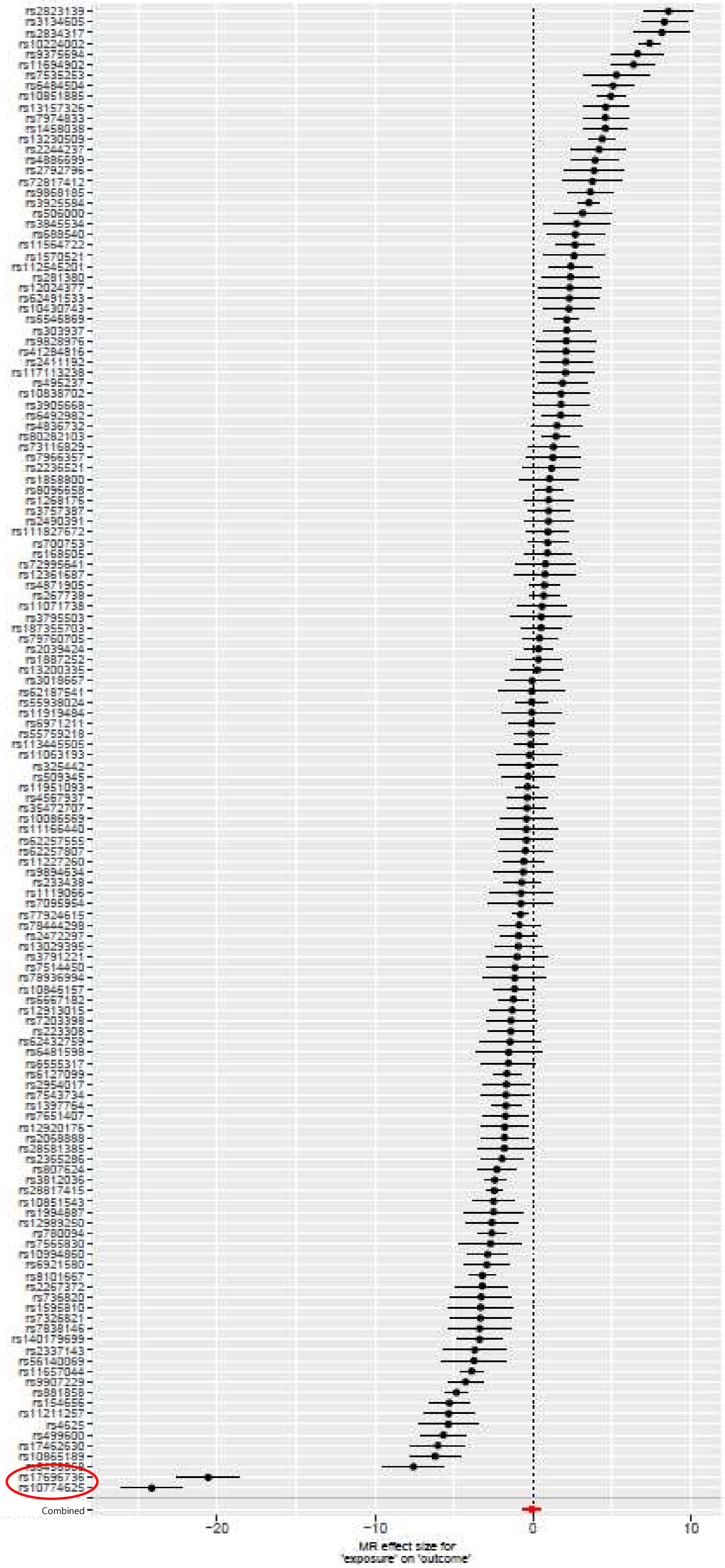

The Wald ratio causal estimates from total 140 SNPs without Steiger processing process to genetically predict eGFR, with an outcome variable, hemoglobin concentration, assessed. Few SNPs that reflect the reverse directional effect (marked in red circle) caused substantial bias to the causal estimates as the SNPs reflect the genetic effects related to the biochemical parameter on eGFR. Thus, Steiger filtering was performed in the main analysis of this study. The error bars indicate 95% confidence interval and the combined effect (red bar) indicates the estimates by inverse variance weighted method.
