# Supplementary material for: Mendelian randomization reveals causal effects of kidney function on various biochemical parameters
Source: Commun Biol. 2022 Jul 18;5:713. doi: 10.1038/s42003-022-03659-4 (PMC9293908; doi:10.1038/s42003-022-03659-4)
Supplement: Supplementary file 2 — Description of Additional Supplementary Files [file 42003_2022_3659_MOESM2_ESM.pdf]

Description of Additional Supplementary Files

File name: Supplementary Data 1
Description: Genetic instrument for kidney function developed from the GWAS metaanalysis within the individuals of European ancestry of the CKDGen data.

File name: Supplementary Data 2
Description: Non-significant causal estimates of 49 biochemical parameters from genetically predicted eGFR.

File name: Supplementary Data 3
Description: : GWAS summary-statistics towards 60 biochemical parameters of 140 SNPs which were instrumented to genetically predict kidney function.
